# Characterization of changes in global gene expression in the hearts and kidneys of transgenic mice overexpressing human angiotensin-converting enzyme 2

**DOI:** 10.1186/s42826-020-00056-y

**Published:** 2020-07-29

**Authors:** Su Hae Lee, Seung Wan Jee, Dae Youn Hwang, Jong Koo Kang

**Affiliations:** 1 Laboratory Animal Resources Division, National Institute of Food and Drug Safety Evaluation, Ministry of Food and Drug Safety, Cheongju 28159, South Korea; 2 Biologics Division National Institute of Food and Drug Safety Evaluation, Ministry of Food and Drug Safety, Cheongju 28159, South Korea; 3 Department of Biomaterials Science, College of Natural Resources & Life Science/Life and Industry Convergence Research Institute, Pusan National University, Miryang 50463, South Korea; 4 College of Veterinary Medicine, Chungbuk National University, Chungju 28644, South Korea

**Keywords:** Human ACE2, Transgenic mice, α-MHC, C57BL/6NKorl, Microarray, Coronavirus

## Abstract

Human angiotensin-converting enzyme 2 (hACE2) has recently received a great attention due to it play a critical role as SARS-CoV receptor in the infection of human body. However, no further analysis for gene regulation has been performed in target tissues of model mice during hACE2 overproduction. To characterize changes in global gene expression in the hearts and kidneys of rtTA/hACE2 double transgenic (dTg) mice in response to hACE2 overexpression, total RNA extracted from these tissues from dTg mice after doxycycline (Dox) treatment was hybridized to oligonucleotide microarrays. Briefly, dTg mice were generated by cross-mating pα-MHC/rtTA Tg mice with pTRE/hACE2 Tg mice. The expression level of hACE2 protein was determined to be high in hearts, kidneys, and brains of dTg mice, whereas lung, liver, and testis tissues expressed low levels. The level of hACE2 was significantly enhanced in hearts and kidneys of the Dox+dTg group compared to that in Vehicle+dTg mice although consistent levels of mouse ACE2 (mACE2) remained in the same tissues. Based on the microarray analysis of heart tissue, 385 genes were differentially expressed, including 168 upregulated and 217 downregulated, when comparing non-Tg and Vehicle+dTg mice, whereas 216 genes were differentially expressed, including 136 upregulated and 80 downregulated, between Vehicle+dTg and Dox+dTg mice. In the kidneys, 402 genes were differentially expressed, including 159 upregulated and 243 downregulated, between non-Tg and Vehicle+dTg mice. Dox-treated dTg mice exhibited the differential expression of 4735 genes including 1636 upregulated and 3109 downregulated. Taken together, these findings suggested that several functional groups and individual genes can be considered biomarkers that respond to hACE2 overexpression in dTg mice. Moreover, our results provided a lot of useful information to predict physiological responses when these dTg mice are applied as a susceptible model for novel coronavirus (SARS-CoV, COVID-19) in both vaccine and drug development.

## Introduction

Angiotensin-converting enzyme 2 (ACE2) is the first known human homolog of ACE and was cloned from a human heart failure and human lymphoma cDNA library [1, 2]. ACE2 might play a pivotal role as a new element of the renin angiotensin system (RAS) by reducing Ang II and increasing levels of Ang1–7 [3–6] and is distributed in a wide variety of tissues, including the brain, lung, heart, liver, kidney, and testis, as well as most cardiovascular-relevant tissues [7–11]. In the heart, myocardial infarction increases ACE2 expression, which is localized to the vascular endothelium, smooth muscle, and cardiomyocytes of both rats and humans [12]. ACE2 participates in the regulation of blood pressure and cardiac and renal functions and is associated with major cardiac and renal pathophysiological processes. Especially, it has been reported that *ACE2* gene expression is upregulated in humans with heart failure [13]. It is also predominantly expressed in the proximal tubular brush border, distal tubules, and glomerular epithelial cells in the kidneys of humans, rats, and mice [14–17]. The colocalization of ACE2 with Ang1–7 in renal tubules reveals the functional ability of Ang1–7 to counteract the action of Ang II, and these vasodilator peptides might be a critical link, mediating regulatory feedback between ACE and ACE2 [18–20]. Although the function of ACE2 in the brain is poorly understood, there is considerable evidence of a role for Ang1–7. Previous studies have shown that Ang1–7 is an important neuromodulator of cardiac baroreflex mechanisms [21]. The ACE1/2 and neutral endopeptidase or neprilysin (NEP) are zinc metallopeptidases [22]. However, more recently, ACE2 has aroused considerable attention as a receptor for the coronavirus that causes severe acute respiratory syndrome and a protector against severe lung failure [23, 24].

In the past decade, functional studies on hACE2 have continued using transgenic animals to clarify the mechanism related to heart and renal failures, as well as other pathophysiological conditions. First, in knockout mice, the genetic disruption of ACE2 leads to severe cardiac contractile dysfunction, increases in Ang II levels, the upregulation of hypoxia-induced genes, and decreases in ACE2 transcript and protein levels in the heart [25]. However, it is necessary to additionally study the effects related to hACE2 expression levels to clarify whether increased levels can have a beneficial effect on cardiac and renal functions.

Myosin heavy chain (MHC), which is found in the contractile apparatus and is a major protein, is composed of two heavy chains and four light chains. In the cardiac muscle, two distinct *MHC* genes, which encode *α-* and *β-MHC* isoforms, have been identified [26]. Previously, the *α-MHC* gene promoter was used to direct tissue- and developmental-specific expression of the transgene in transgenic (Tg) animals [27]. Further, there are many transcriptional regulation systems, and tetracycline regulatory systems have been widely used for conditional gene expression. The tetracycline-controlled transactivator (tTA) is generated by fusing the DNA-binding domain of the tetracycline-resistance operon (TetR) encoded by Tn10 of *E. coli* with the transcriptional activation domain of VP16 of herpes simplex virus [28]. There are two basic variants of this, tTA and reverse tTA (rtTA) systems. To compensate for the tTA system, which requires long-term administration of doxycycline (dox) for induction of the transgene, the other inducible system, namely rtTA (Tet-On system), has been developed. This system requires two DNA constructs, a transcriptional regulatory unit and the responsive element *tetO* sequences linked to a P_CMV_-derived target gene. In the presence of dox, rtTA binds *tetO* sequences and P_CMV_, which activates the target gene. In contrast, rtTA does not bind *tetO* and the target gene is not transcribed in the absence of dox [29, 30]. The dox-inducible gene regulatory system allows for the tight and adjustable control of a transgene of interest to study organ development and disease pathogenesis. Previously, Tg rats expressing human *ACE* under the control of the rat cardiac *myosin light chain 2 (MLC2)* promoter and Tg mice expressing human *ACE2* under the control of the mouse cardiac *α-MHC* promoter have been generated [31, 32]. These Tg mice had a high incidence of sudden death and rhythmic disturbances with sustained ventricular tachycardia including terminal ventricular fibrillation and heart block. However, in surviving older mice, spontaneous downregulation of the *ACE2* transgene was observed, which was associated with the restoration of nearly normal conduction, rhythm, and connexin expression. Because sudden death occurs earlier in higher-expressing transgenic lines, a promoter that can be regulated, such as the Tet-On or Off system, might be useful to examine cardiac and renal pathophysiology at the basal level. The aim of this study was to characterize changes in global gene expression in the hearts and kidneys of dTg mice, created with pα-MHC/rtTA and pTRE/hACE2 vectors, in response to the overproduction of hACE2 protein.

## Materials and methods

### Construction of dox-inducible vector system

**Fig. 1:**
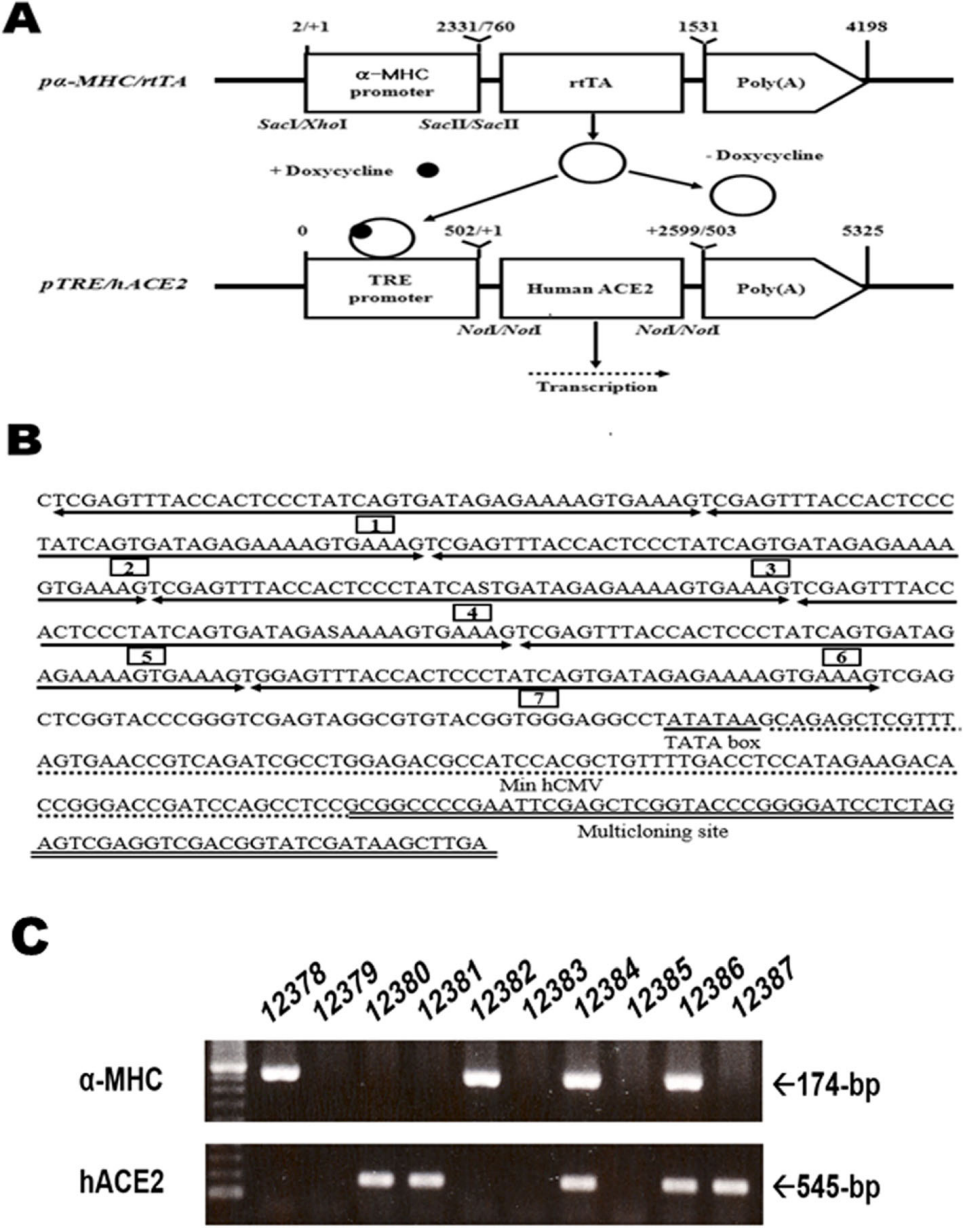
Reverse *tet* promoter-controlled transactivator (rtTA) and hACE2 vectors and identification of *α-MHC/rtTA* and *TRE/hACE2* transgenes. **a** Construction of the *pα-MHC/rtTA* and *pTRE/hACE2* expression vectors. *rtTA* and *hACE2* (human ACE2) were placed under the control of the *α-MHC* and *TRE* promoter, respectively. Two independent lineages of transgenic (Tg) mice were produced and mated together to obtain the double Tg (dTg) mice. In the dTg mice, the expression of the *hACE2* is induced by *rtTA* in the presence of doxycycline (Dox). The arrow (--->) indicates transcription. **b** Features of the *tet* response element (TRE) promoter sequence. The TRE promoter is contained with seven copies of the *tetO* sequence, a TATA box, and an hCMV promoter. **c** The genomic DNA was isolated from the tail of the founder mouse, and the 174-bp and 545-bp products were shown in the dual transgenic mice carrying the *α-MHC/rtTA* and *TRE/hACE2* transgenes, respectively

First, *pα-MHC/rtTA* was constructed by fusing the *rtTA-M2* gene with the *α-MHC* promoter (Gene bank accession NO. U71441). The *pUHrT62–1* was a gift from Dr. Wolfgang Hillen at the University of Heidelberg, Germany [33]. This plasmid contains a mutagenized *rtTA-M2* fragment harboring the S12G, E19G, and A56P mutations. The *α-MHC* sequence was amplified by PCR, with the genomic DNA as a template, which was isolated from the tail of BDF2 mice. The following primers were used for the amplification: the sense primer, 5′-CTCCT TCCTT GTTGC ATCTT CC-3′ (corresponding to nucleotides 2,278–2,299 of *α-MHC*), and the antisense primer, 5′-CAGGA GGAAG ATGGA GAAGA CAG-3′ (corresponding to nucleotides 4,578–4,600 of *α-MHC*). The amplified *α-MHC* product was cloned into *pGEM-T* (*pα-MHC-T)*. The *α-MHC* fragment obtained by the digestion of *pα-MHC-T* with *Sac*I and *Sac*II was cloned into *pUHDrtTA2S-M2-splice*, in which the human *CMV* promoter has been eliminated via digestion with *Xho*I *and Sac*II (*pa-MHC/rtTA)*. Second, *pTRE/hACE2* was constructed by inserting the *hACE2* gene into the *Not*I site within the multiple cloning site of *pTRE2hyp* (Clontech Laboratories Inc., Mountain View, CA, USA). The *pTRE2hyp* gene contains a *tet* response element (TRE), which consists of seven copies of the 19-bp tetracycline operator sequence (*tetO*) and hygromycin resistance genes. The *hACE2* cDNA was amplified by PCR, using a sense primer (5′-GACGA TGTCA AGCTC TTCCT G-3′) with nucleotides 100–120 and an antisense primer (5′-GCCTA cagat cttct tcaga aataa gtttt tgttc AAAGG TCTGA ACATC ATCAG TG-3′; lowercase letter, *c-myc* tag) with nucleotides 2,494–2,521 based on *hACE2* (Gene bank accession NO. NM_021804). Full-length RNA was used as the template, which was isolated from HEK-293 cells (ATCC CRL-1573). The amplified *hACE2* product was inserted into *pGEM-T* (*phACE2-T*; Fig. 1a and b).

### Maintenance of experimental animals

The experimental protocol for dTg mice was carefully reviewed based on ethical and scientific care guidelines and approved by the National Institute of Food and Drug Safety Evaluation-Institutional Animal Care and Use Committee (NIFDS-IACUC; Approval No. NITR0631). All C57BL/6NKorl and DBA/2Korl mice at 6 weeks of age were provided by the Department of Laboratory Animals Resources in NIFDS (Cheongju, Korea). All mice used in this study were provided with *ad libitum *access to water and an irradiated standard chow diet (Purina Mills Inc., Pyeongtaek, Korea). Mice were housed in cages under specified pathogen free (SPF) condition under a strict light cycle (light on at 06:00 h and off at 18:00 h). All mice were handled in an accredited NIFDS animal facility in accordance with the AAALAC International Animal Care policies (Accredited Unit-the Ministry of Food and Drug Safety: Unit Number-001492).

### Production of rtTA/hACE2 dTg mice

For the first lineage of Tg mice, a linear 5.8-kb *α-MHC/rtTA* fragment was microinjected into the pronucleus of fertilized eggs of BDF1 mice, which had been obtained by mating C57BL/6NKorl (males) and DBA2Korl (females) mice. For the second lineage of Tg mice, the linear 7.9-kb fragment of *TRE/hACE2* was microinjected into the pronucleus of a fertilized embryo after dilution to a concentration of 4 ng/μL. Microinjection was performed using microscopy (Nikon, Tokyo, Japan) and a micromanipulator (Narishge, Tokyo, Japan). Each Tg line was established by back crossing the founder mice with a parental strain of C57BL/6NKorl mice. The dTg mice were obtained by crossing the first lineage of *α-MHC/rtTA* Tg mice with the second lineage of TRE/hACE2 Tg mice. The single and double Tg founder mice were back crossed onto the parental strain of the C57BL/6NKorl background to establish homogenous Tg lines.

The transgene was identified by DNA-PCR analysis of the genomic DNA isolated from the tail of 4-week-old founder mice. The precipitated DNA was separated by centrifugation at 15,000 rpm for 10 min. After washing in 700 μL 70% Et-OH, DNA was dried for 15 min and dissolved in 200 μL of distilled water. The *α-MHC/rtTA* gene was amplified as a template using the sense primer (5′-CTGTC TTCTC CATCT TCCTC CTG-3′) with a complementary *α-MHC* promoter ranging from 4578 to 4600 nucleotides and an antisense primer (5′-CAGGG TAGGC TGCTC AACTC-3′) with a complementary *rtTA* gene ranging from 888 to 908 nucleotides. The *TRE/hACE2* gene was also synthesized as a template using a sense primer (5′-GACGA TGTCA AGCTC TTCCT G-3′) and antisense primer (5′-CATAT AATGG CCTCA GCTGC-3′) with a complementary *hACE2* gene ranging from 100 to 120 and from 625 to 644 nucleotides, respectively. PCR amplification was carried out in a Thermal cycler (Perkin Elmer, Norwalk, CT, USA) using the following cycling conditions: one cycle: 94°C, 4 min; 27 cycles: 30 s at 94°C, 1 min at 62 °C, and 45 s at 72°C; one elongation step of 7 min at 72°C.

### Doxycycline (dox) treatment

The dTg mice were distributed into two groups (3–5 mice per group), namely vehicle and Dox treatment. Drinking water containing 2 mg/mL Dox (Sigma-Aldrich Co., St. Louis, MO, USA) was administered to mice of the Dox-treated group for 4 weeks, whereas tap water was administrated to mice of the vehicle group for the same period.

### Western blot analysis

The brain, heart, lung, liver, kidney, and testis tissues were collected from mice of subset groups and homogenized with 1% nnonidet P-40 in 15 mM NaCl, 10 mM tris HCl, and 1 mM EDTA supplemented with a protein inhibitor mixture (Roche, Basel, Switzerland), which was followed by centrifugation at 4°C for 15 min. For western blot analysis, the proteins were separated by electrophoresis using a 4–20% gradient SDS-polyacrylamide gel for 2 h and then transferred to a nitrocellulose membrane with transfer buffer containing 25 mM tris-base, 192 mM glycine, and 20% methanol at 45 V for 2 h. Membranes were blocked with 3% (w/v) non-fat dried milk in PBS (137 mM NaCl, 2.7 mM KCL, 10 mM Na_2_HPO and 2 mM KH_2_PO_4_) solution containing 0.05% tween-20 (Sigma-Aldrich Co.). The membrane was washed with PBS and incubated overnight 4°C with primary antibodies as follows: anti-human ACE2 (Santa Cruz Biotechnology Inc., Santa Cruz, CA, USA), anti-human ACE (Santa Cruz Biotechnology Inc.), and anti-α-tubulin (Sigma-Aldrich Co.). After washing, each antigen–antibody complex was visualized with biotinylated secondary antibodies as follows: goat anti-mouse IgG (H+L) HRP-conjugated antibody, rabbit anti-goat IgG (H+L) HRP-conjugated antibody, and goat anti-rabbit IgG (H+L) HRP-conjugated antibody (Zymed Laboratories Inc., San Francisco, CA, USA) at a 1:1000 dilution in PBS buffer containing 3% non-fat dried milk/PBS buffer at room temperature for 1 h. Immunoreactive proteins were detected by an enhanced chemiluminescent substrate (ECL, Amersham Pharmacia Biotech, Inc., Amersham, England) reaction, which was followed by exposing the membranes to hyperfilm ECL.

### RNA isolation for microarray analysis

The hearts and kidneys from non-Tg, Vehicle+dTg, and Dox+dTg mice were used for the isolation of total RNA using RNAzol (Tel-Test Inc., Friendswood, Texas, USA) according to the manufacturer’s instructions. The frozen tissues were minced with scissors and homogenized in RNAzol B solution using a Teflon glass homogenizer. The RNA pellet was suspended in DEPC-treated dH_2_O and purified using a QIAquick purification kit (Qiagen Inc., Chatsworth, CA, USA). The integrity of the 18S/28S rRNA was analyzed using a Bioanalyzer 2100 (Quality Agilent Technology Inc., Santa Clara, CA, USA), with the RNA quality checked based on the ratio of absorbance at 260 nm to that at 280 nm using a Biophotometer (Hamburg-Eppendorf, Hamburg, Germany). The integrity of each RNA sample was confirmed by 1% agarose gel electrophoresis, which showed the presence of intact 18S and 28S ribosomal bands.

cRNA synthesis and labeling were performed using a Chemiluminescent RT-IVT Labeling Kit (Applied Biosystems, Foster City, CA, USA) according to the manufacturer’s instruction. Individual samples were submitted in randomly assigned pairs representing tissues from non-Tg and Tg mice. Three micrograms of total RNA was used to synthesize cDNA. Double stranded cDNA was synthesized for 2 h at 16°C in a reaction mixture containing nuclease free water, 5× 2^nd^ strand buffer, and 2^nd^ strand enzyme and then purified using a purification column. Finally, the biotinylated cRNA was generated from the cDNA using a BioArray cRNA High Yield RNA Transcript Kit containing purified double stranded cDNA, 5× IVT buffer Mix, DIG-UTP and IVT enzyme mix.

### Microarray analysis

Changes in global gene expression in the hearts and kidneys of mice overexpressing human *ACE2* were analyzed using the Mouse Genome Survey Microarray (Applied Biosystems) containing oligonucleotide probes for 44,000 genes. The cRNA was fragmented in fragmentation buffer for 30 min at 60°C prior to chip hybridization. Fifteen micrograms of fragmented cRNA was then added to the hybridization cocktail. Each sample was hybridized to a separate oligonucleotide array for 16 h at 55°C in the GeneChip Hybridization Oven 640. After hybridization, the solution was removed and the slides were washed twice with 2× SSC containing 0.1% SDS for 5 min at 42°C. The slides were incubated for 20 min with anti-Dig-AP in CL blocking buffer and then developed using the chemiluminescence substrate. Thereafter, the hybridized arrays were scanned using an ABI700 analyzer. Scanning and basic analyses were performed using GenePlex software release 1.0 (ISTECH Inc., Seoul, Korea). Logged gene expression ratios from the fluorescent intensity of each spot were normalized based on regression.

### Statistical analysis

Tests for significance were performed using a one-way ANOVA test of variance (SPSS for Windows, Release 10.01, Standard Version, and Chicago, IL, USA). All values are reported as the mean ± standard deviation (SD). Microarray data and the statistical significance of differential expression were assessed by hypergeometric distribution analysis. *P* values less than 0.05 were considered significant.

## Results

### Establishment of dTg mice

First, we produced one male founder mouse carrying the *pα-MHC/rtTA* construct and one female founder mouse carrying the *TRE/hACE2* construct using microinjection techniques. These showed different rates of transgene insertion into the chromosome at 18 and 7%, respectively. Moreover, *α-MHC/rtTA* and *TRE/hACE2* single Tg mice with a BDF2 background were backcrossed with C57BL/6NKorl mice to change their background. During this process, *α-MHC/rtTA* and *TRE/hACE2* constructs were transmitted into the genomes of their offspring of both sexes at a rate of approximately 50% hemizygous animals based on Mendelian inheritance. Furthermore, both construct genes were transmitted into the genomes of dTg mice at a 16.3% efficiency.

### Tissue-specific expression of hACE2 during dox treatment

**Fig. 2:**
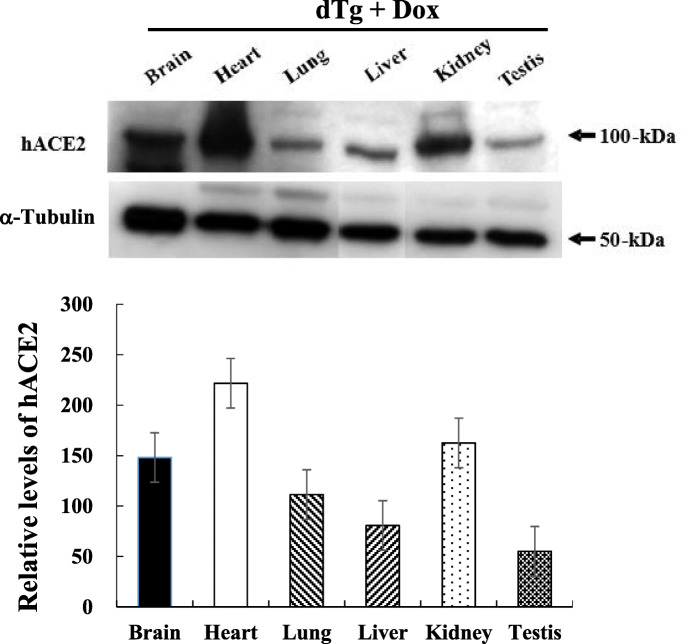
Human ACE2 (hACE2) levels in organs of double transgenic (dTg) mice treated with doxycycline (Dox). Proteins isolated from the tissues were analyzed by western blotting using an anti-hACE2 antibody, and the α-tubulin signal was used as a control. The density of the protein bands was quantified by Image Station 2000MM. The values represent the mean ± SD

To test whether the *hACE2* transgene was expressed under the control of rtTA in a tissue-specific manner, its expression level was detected in various tissues including the brain, heart, lung, liver, kidney, and testis of dTg mice treated with 2 mg/mL Dox for 4 weeks. The expression level of *hACE2* was higher in the heart, kidney, and brain than in other organs, although the highest level was detected in the heart. A low level of hACE2 protein expression was observed in the lung, liver, and testis (Fig. 2). However, any significant expression of hACE2 protein was not detected in heart and kidney of non-Tg and C57BL/6 mice (data not shown). Therefore, these results indicate that the hACE2 protein might be successfully expressed in various tissues of dTg mice through regulation of the Dox-controlled rtTA system.

### Comparison of mACE and hACE2 expression in the heart and kidney

**Fig. 3:**
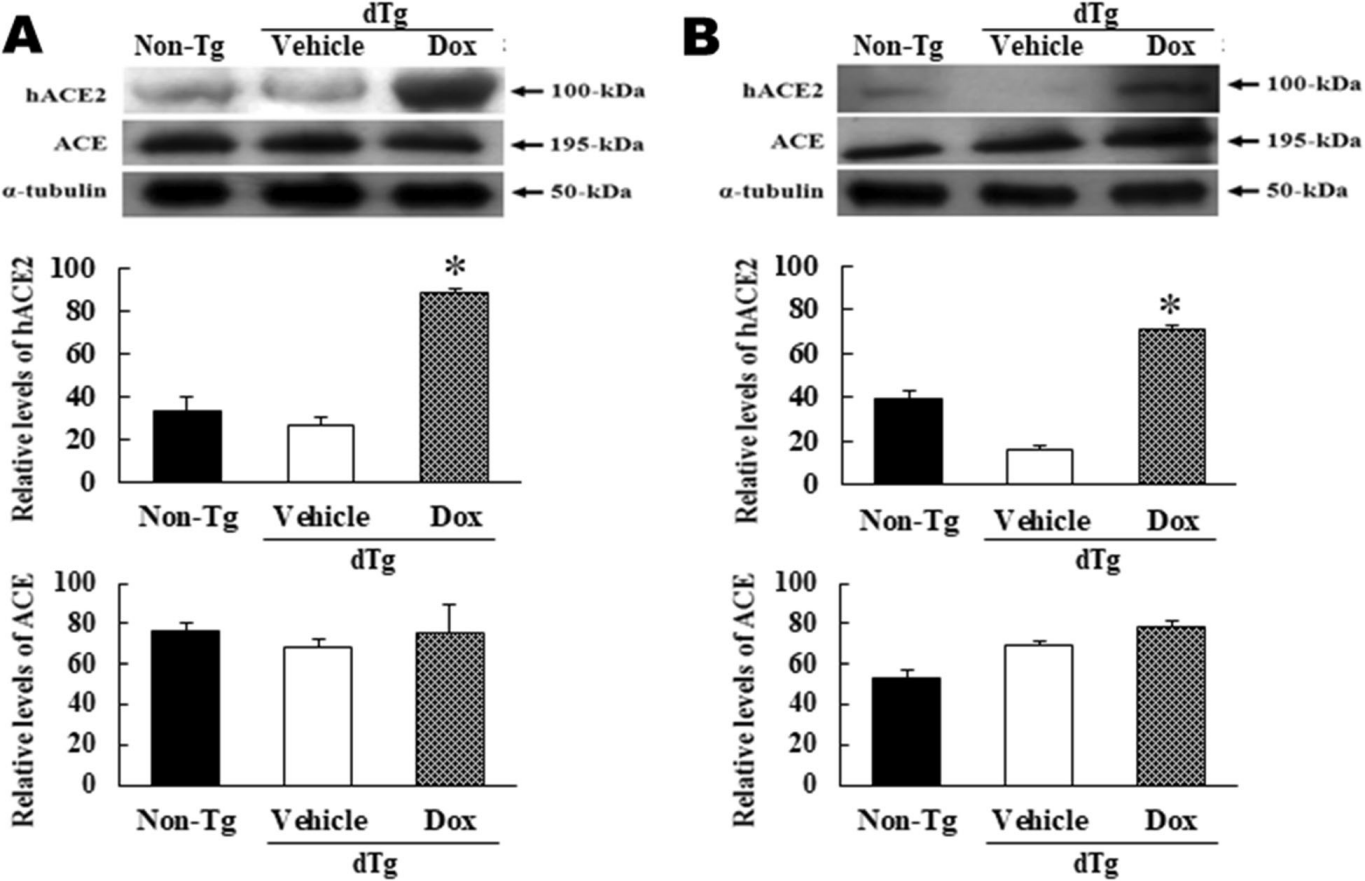
ACE (mouse) and human ACE2 (hACE2) levels in organs of doxycycline (Dox)-treated dTg mice. Shown are protein levels in hearts (**a**) and kidneys (**b**). The proteins isolated from the tissues of the Tg mice treated with Dox were analyzed by western blotting using an anti-ACE and anti-hACE2 antibody for each protein. The α-tubulin signal was used as a control. The values represent the mean ± SD. * *p* < 0.05 compared with the Vehicle+dTg group. Abbreviations: non-Tg, non-transgenic mice; dTg, double transgenic mice

To compare the expression level of mACE and hACE2 proteins in the heart and kidney with or without Dox treatment, both proteins were detected with specific antibodies in these tissues of non-Tg, Vehicle+dTg, and Dox+dTg mice at the age of 10 weeks. A similar expression pattern for both proteins was observed in the heart and kidney. The *hACE2* gene was highly expressed in only Dox+dTg mice, whereas in non-Tg and Vehicle+dTg groups, expression was maintained at low level. However, the expression level of mACE protein remained constant regardless of hACE2 expression and Dox treatment (Fig. 3a and b). Thus, these results suggest that hACE2 protein can be successfully overexpressed in the hearts and kidneys of dTg mice after Dox treatment. Further, it was suggested that these organs would have great potential for microarray analysis to characterize the changes in global genes during overexpression of the hACE2 protein.

### Ontology categories of hACE2-regulated gene expression

To analyze the differential expression of global genes during hACE2 overproduction in the heart and kidney, microarray data were normalized based on the lowest values obtained from the experiments repeated in triplicate. The normalized log ratios of various genes in each experiment were used to compare relative expression between two independent experiments. Based on these arbitrary differences, a substantial number of genes were expressed at elevated or reduced levels in the Tg group before and after dox treatment. In the heart, 385 transcripts were selected as differentially expressed genes between non-Tg and Vehicle+dTg groups based on a 2-fold change in expression, whereas 216 differential transcripts were identified between Vehicle+dTg and Dox+dTg groups. Among the former 385 genes, 168 were upregulated and 217 were downregulated in the heart tissue of the dTg group as compared to levels in the non-Tg group before Dox treatment. Moreover, 216 genes, including 136 upregulated and 80 downregulated, were differentially expressed in the dTg group after Dox treatment (Table 1). In the kidney, a total of 402 genes, including 159 upregulated and 243 downregulated genes, were changed in the Vehicle+dTg mice compared to levels in non-Tg mice. Furthermore, after Dox treatment for 4 weeks, 4735 genes, comprising 1636 upregulated and 3109 downregulated, were altered in the dTg group based on a 3-fold change in expression (Table 1). Overall, these results suggest that hACE2 overproduction is more closely related to differentially expressed genes in the kidney than in the heart.

**Table 1:** Summary of differentially expressed genes in Vehicle-treated dTg mice and Dox-treated dTg mice

Category	Non-Tg vs Vehicle+dTg	Vehicle+dTg vs Dxo+dTg
**44 K Whole Mouse Genome**		
**(Agilent)**	44,000	44,000
**Heart**		
Differential expression of known genes at the cut-off ratio of > 2-fold	Total genes, *n* = 385 Upregulated genes, *n* = 168 Downregulated genes, *n* = 217	Total genes, *n* = 216 Upregulated genes, *n* = 136 Downregulated genes, *n* = 80
**Kidney**		
Differential expression of known genes at the cut-off ratio of > 3-fold	Total genes, *n* = 402 Upregulated genes, *n* = 159 Downregulated genes, *n* = 243	Total genes, *n* = 4735 Upregulated genes, *n* = 1636 Downregulated genes, *n* = 3109

Abbreviations: *non-Tg* Non-transgenic mice, *dTg* Double transgenic mice, *Dox* Doxycycline

### KEGG pathway analysis of hACE2-regulated gene expression

To analyze the KEGG pathways associated with hACE2-regulated gene expression in the heart and kidney of non-Tg and dTg mice based on Dox treatment, GenPlex software release 1.0 was used. The results are presented in Tables 2 and 3. GenPlex identified 15 pathways that were associated with significant changes in the heart (Table 2). As presented in Table 2, the largest numbers of genes in the heart were mainly associated with cytokine-cytokine receptor interaction, the calcium signaling pathway, apoptosis, and ECM-receptor interaction. In the kidney, 17 pathways were identified (Table 3). Especially, genes involved in Wnt signaling were highly changed, followed by those related to oxidative phosphorylation, prostate and colorectal cancer, the TGF-beta signaling pathway, and apoptosis.

**Table 2:** Gene ontology and KEGG pathways related to human ACE2 overexpression in hearts of dTg mice

***P***-value	KEGG pathway	Gene counts
3.70E-04	ECM-receptor interaction	8
5.55E-04	Metabolism of xenobiotics by cytochrome P450	7
0.00359981	Arachidonic acid metabolism	6
0.01443938	Type II diabetes mellitus	4
0.02960078	Cytokine-cytokine receptor interaction	31
0.030258432	TGF-beta signaling pathway	5
0.03164412	Glycolysis / Gluconeogenesis	4
0.033455342	Starch and sucrose metabolism	4
0.049109556	One carbon pool by folate	2
0.002257311	Apoptosis	10
0.013300485	Nitrogen metabolism	4
0.016855394	Calcium signaling pathway	14
0.0169800262	Fatty acid biosynthesis	2
0.023873353	Hematopoietic cell lineage	8
0.03435755	Amyotrophic lateral sclerosis (ALS)	3

Mice were treated with doxycycline (Dox) for 4 weeks; dTg = double transgenic

**Table 3:** Gene ontology and KEGG pathways related to human ACE2 overexpression in kidneys of dTg mice

P-value	KEGG pathway	Gene counts
2.12E-11	Oxidative phosphorylation	97
0.001377066	Valine, leucine and isoleucine degradation	35
0.006218532	Prostate cancer	78
0.009537098	One carbon pool by folate	17
0.017347632	Glycerolipid metabolism	34
0.018135441	Amyotrophic lateral sclerosis (ALS)	19
0.021784257	Basal cell carcinoma	41
0.023491979	DNA polymerase	23
0.026797164	1- and 2-Methylnaphthalene degradation	20
0.028321892	Ubiquinone biosynthesis	8
0.029679868	TGF-beta signaling pathway	67
0.032515198	Colorectal cancer	73
0.032849174	Wnt signaling pathway	110
0.038628325	Antigen processing and presentation	49
0.04013507	Apoptosis	65
0.045726832	Fatty acid metabolism	29
0.048242938	Pentose and glucuronate interconversions	13

Mice were treated with doxycycline (Dox) for 4 weeks; dTg = double transgenic

Furthermore, we characterized the genes that were downregulated and upregulated by hACE2 overproduction in the hearts and kidneys of Dox+dTg mice. Of the 136 upregulated genes in the heart, the highest difference was detected for *Cap1*, followed by *Pik3c2g*, *Syt16*, *Hecw1*, and *Tmod2*, whereas downregulated genes included *calgranulin A*, *calgranulin B*, *Paip1*, *Ptx3*, and *Hspa1a*. In the kidney, *Gabrg1* was associated with the highest increase, followed by *Wdr66*, *Ppp1r14c*, *Bin1*, and *Mef2c* among 1636 upregulated genes, but *Slc16a4*, *Fgfr3*, *Slc16a10*, *Stam2*, and *Mrps2* were significantly decreased after hACE2 overproduction. Taken together, this showed that hACE2 overexpression is mainly associated with cellular pathways related to cytokine-cytokine receptor interactions, the calcium signaling pathway, apoptosis, ECM-receptor interactions, Wnt and TGF-beta signaling, cancer, and oxidative phosphorylation in the hearts and kidneys of dTg mice.

## Discussion

Recently, advances in molecular biology have enabled functional analyses of interesting genes by using transgenic animals and stable cell lines with constant gene expression. However, if the transgene is related to the embryogenesis, the animal might be genetically predisposed to tolerate the effects of the transgene products [34]. To overcome this problem, it is necessary to develop transgenic animals in which transgene expression can be induced at selected time points but kept silent for an extended period. Conditional gene expression has been achieved using a variety of model systems [35, 36]. Non-regulatable promoters in Tg animals cannot direct the genetic switches to upregulate or downregulate expression from the promoter-linked target gene, and therefore, it is impossible to know how the target gene interacts and regulates the pathophysiological processes at both the basal and inducible level. The Tet-On and Tet-Off expression systems are the most widely used inducible regulatory systems. In the Tet-On system, the reverse tetracycline-controlled transactivator (rtTA) acts as an activator of gene transcription [29, 37]. Dox binds the dox-binding site of the rtTA protein, which represents random mutagenesis of the tTA fusion protein between the Tet repressor DNA-binding domain (207 amino acids) and the VP-16 transcriptional activation domain (130 amino acids) of the herpes simplex virus. The dox–rtTA complex then binds the *tet* sequence, which brings the VP-16 activation domain in close proximity to the minimal human CMV promoter, thereby activating the target gene in the presence of Dox. Indeed, rtTA under the control of the CMV promoter was previously shown to activate the expression of a target gene in various organs [38, 39]. In this study, the inducible Tg mice expressing α-MHC-controlled, rtTA-regulated hACE2 were generated to address the hypothesis that unregulated expression of the h*ACE2* transgene leads to the generation of defects including cardiac contractile dysfunction, rhythmic disorders, and sudden death. First, single Tg mice expressing α-MHC-controlled rtTA and hACE2 were successfully developed by directly introducing each gene into fertilized eggs. Tg mice were mated to induce rtTA-regulated hACE2 expression, which generated founder mice in which hACE2 expression could be increased by Dox. In the established Tg line, neither the location of the transgenes in the genome nor the locus was impacted by gestation or neonatal imprinting. Because of this, all offspring in this experiment exhibited the transmission of *α-MHC/rtTA* and *TRE/hACE2* genes into their genomes in approximately 50% of hemizygotes.

ACE is considered the central enzyme in the RAS, converting Ang I to Ang II. However, the identification of novel RAS components such as ACE2 and collectrin, a homologue of ACE, capable of degrading Ang II and forming Ang1–7, has emphasized the increasing complexity and multiplicity of biochemical pathways forming the RAS. In a previous study, non-regulatable promoters have been used to create transgenic rats or mice expressing *ACE* or *ACE2* genes under control of the rat cardiac *MLC2* or mouse cardiac *α-MHC* promoter [7, 32]. Unexpectedly, the loss of ACE2 in mice results in profound contractile dysfunction. However, the complete rescue of the heart phenotype in ACE/ACE2-double mutant mice indicates that ACE expression has a causative role in the onset of heart dysfunction [25]. Because ACE2 is expressed in the vascular endothelium and not in cardiac myocytes, local increases in Ang II might lead to vasoconstriction, resulting in hyperperfusion and hypoxia in the myocardium. It has been established that Ang II can induce oxidative stress in endothelial cells, and thus, its increase could result in dysfunction of the vascular endothelium via the induction of oxidative stress in the heart [25, 40, 41]. In this study, a heart-specific promoter system was employed to induce rtTA-regulated hACE2 expression at a physiologically relevant site. hACE2 protein was abundantly expressed in heart and kidney tissue of Dox+dTg mice, although this protein was also detected in several other tissues. These results suggest that the binding of Dox to the rtTA protein might successfully induce hACE2 expression in the heart, kidney, and other organs of dTg mice. However, any significant histopathological changes was not observed in heart and kidney of Dox+dTg mice after induction of hACE2 expression for only 4 weeks (Supplement Fig. 1).

Interestingly, the RAS can be seen as a dual function system in which vasoconstrictor or vasodilator actions are primarily driven by the ACE/ACE2 balance [42–44]. Elevated ACE2 activity concomitant with reduced ACE activity leads to a decrease in Ang II levels by converting it into Ang1–7, which in turn promotes vasodilatation [45]. According to this concept, Dox-driven hACE2 expression resulted in a decrease in ACE levels in both hearts and kidneys of Dox-inducible Tg mice compared to that in Non-Tg mice. These results imply that the effects of hACE2 expression, via the formation of Ang1–7, have a counter regulatory role in ACE activity in heart and kidney functions.

The present study also investigated gene profiles to offer critical insight into complexity of the RAS, as well as heart and renal failure related to hACE2 overexpression. Inducible Tg mice overexpressing hACE2 and non-Tg mice were used to address the hypothesis that genes many genes involved in cardiovascular and renal disorders are modulated as compared to levels in Dox+dTg and Non-Tg mice. The results identified 136 genes from the hearts of Dox+dTg mice that were significantly upregulated and 80 genes that were downregulated compared to levels in the hearts of Vehicle+dTg mice. A total of 216 genes associated with cellular pathways comprising 15 categories mainly related to ECM-receptor interaction, cytokine-cytokine receptor interaction, apoptosis, the calcium signaling pathway, and the TGF-beta signaling pathway were identified. Of these genes, *S100a8* and *S100a9*, encoding S100 calcium-binding proteins that bind several types of proinflammatory cytokines, such as TNF-alpha, IL-6, and IL1beta, to form the proinflammatory cytokine complex in acute inflammation, play a role in calcium-mediated signaling [46, 47]. *Ptx3*, a pentraxin-related gene, is induced in vascular smooth muscle cells via atherogenic modified low density [48] and acts as a nonredundant regulator of tissue damage in acute myocardial ischemia and reperfusion [49]. Thus, these results suggest that the overexpression of hACE2 might be involved in acute myocardial infarction and ischemia in the hearts of inducible rtTA-regulated Tg mice. In the kidney, 1636 genes in Dox-treated Tg mice were upregulated and 3109 genes were downregulated. These genes were also associated with cellular pathways comprising 17 categories related to apoptosis, the calcium and Wnt signaling pathway, oxidative phosphorylation, cancer, and the TGF-beta signaling pathway. Especially, hACE2 expression was mainly associated with the cellular pathway related to cytokine-cytokine receptor interactions, the calcium signaling pathway, apoptosis, ECM-receptor interactions, Wnt and TGF-beta signaling, cancer, and oxidative phosphorylation, and rtTA-controlled hACE2 expression regulates the expression of genes related to binding, receptor, transferase, oxidoreductase, and hydrolase activities in the hearts and kidneys of dTg mice. However, our study provides limited information since only mRNA level were analyzed to characterize global gene expression in response to hACE2 overexpression. Furthermore, protein expression analyses and molecular mechanism studies are necessary to conform the effects of identified genes in future study.

## Conclusions

Taken together, we produced rtTA/hACE2 dTg mice that overexpress the *hACE2* gene based on a Dox-inducible system and analyzed the changes in global gene expression in heart and kidney tissue using a microarray. The results of the present study suggest that hACE2 protein was successfully expressed in the heart, kidney, and brain of dTg mice after Dox treatment. Moreover, our microarray analysis was able to identify several functional groups of genes and individual genes that respond to hACE2 overexpression in the hearts and kidneys of Tg mice. However, additional work should address the extent to which these changes are correlated with hACE2 expression levels to determine whether beneficial effects can be obtained by reducing expression levels or if the increased expression of ACE2 at any level is deleterious with respect to cardiac and renal disease. Furthermore, it will be necessary to study the functions of differentially expressed genes, which could represent targets for the development of novel drugs, using pharmacoproteomics.

## Supplementary information

**Additional file 1.**

**Additional file 2.**

**Additional file 3.**

**Additional file 4.**

## Data Availability

Available.

## Abbreviations

ACE2: Angiotensin-converting enzyme 2
MHC: Myosin heavy chain
rtTA: Reverse tetracycline-controlled transactivator
TetR: Tetracycline- resistance operon
TRE: *Tet* response element
