# Supplementary material for: Characterization of changes in global gene expression in the hearts and kidneys of transgenic mice overexpressing human angiotensin-converting enzyme 2
Source: Lab Anim Res. 2020 Jul 29;36:23. doi: 10.1186/s42826-020-00056-y (PMC7387885; doi:10.1186/s42826-020-00056-y)
Supplement: Supplementary file 1 — Additional file 1. [file 42826_2020_56_MOESM1_ESM.pptx]

## Slide 1
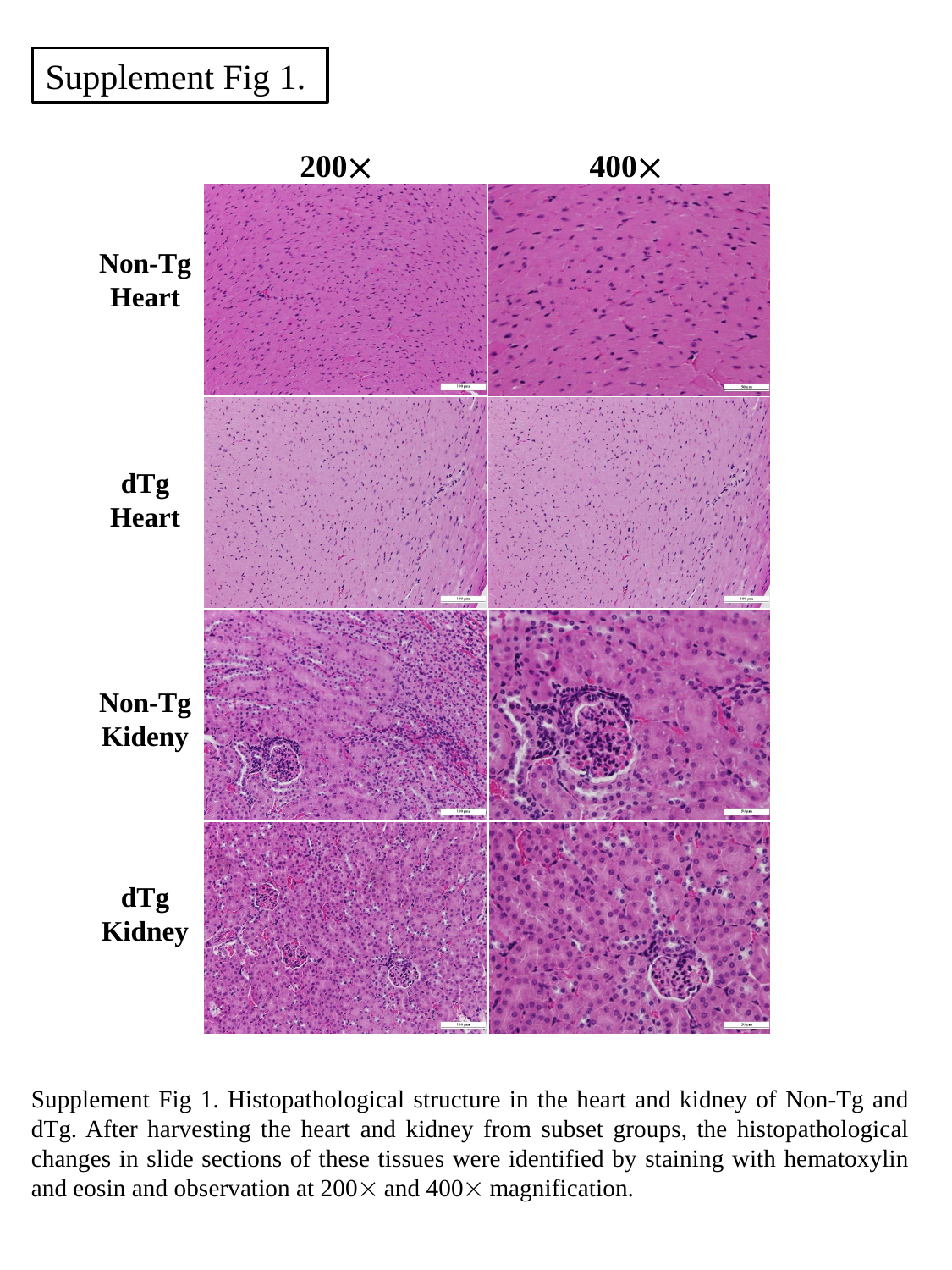

Supplement Fig 1.
200× 400×
Non-Tg
Heart
dTg
Heart
Non-Tg
Kideny
dTg
Kidney
Supplement Fig 1. Histopathological structure in the heart and kidney of Non-Tg and dTg. After harvesting the heart and kidney from subset groups, the histopathological changes in slide sections of these tissues were identified by staining with hematoxylin and eosin and observation at 200× and 400× magnification.
