# Supplementary material for: Characterization of changes in global gene expression in the hearts and kidneys of transgenic mice overexpressing human angiotensin-converting enzyme 2
Source: Lab Anim Res. 2020 Jul 29;36:23. doi: 10.1186/s42826-020-00056-y (PMC7387885; doi:10.1186/s42826-020-00056-y)
Supplement: Supplementary file 2 — Additional file 2. [file 42826_2020_56_MOESM2_ESM.pptx]

## Slide 1
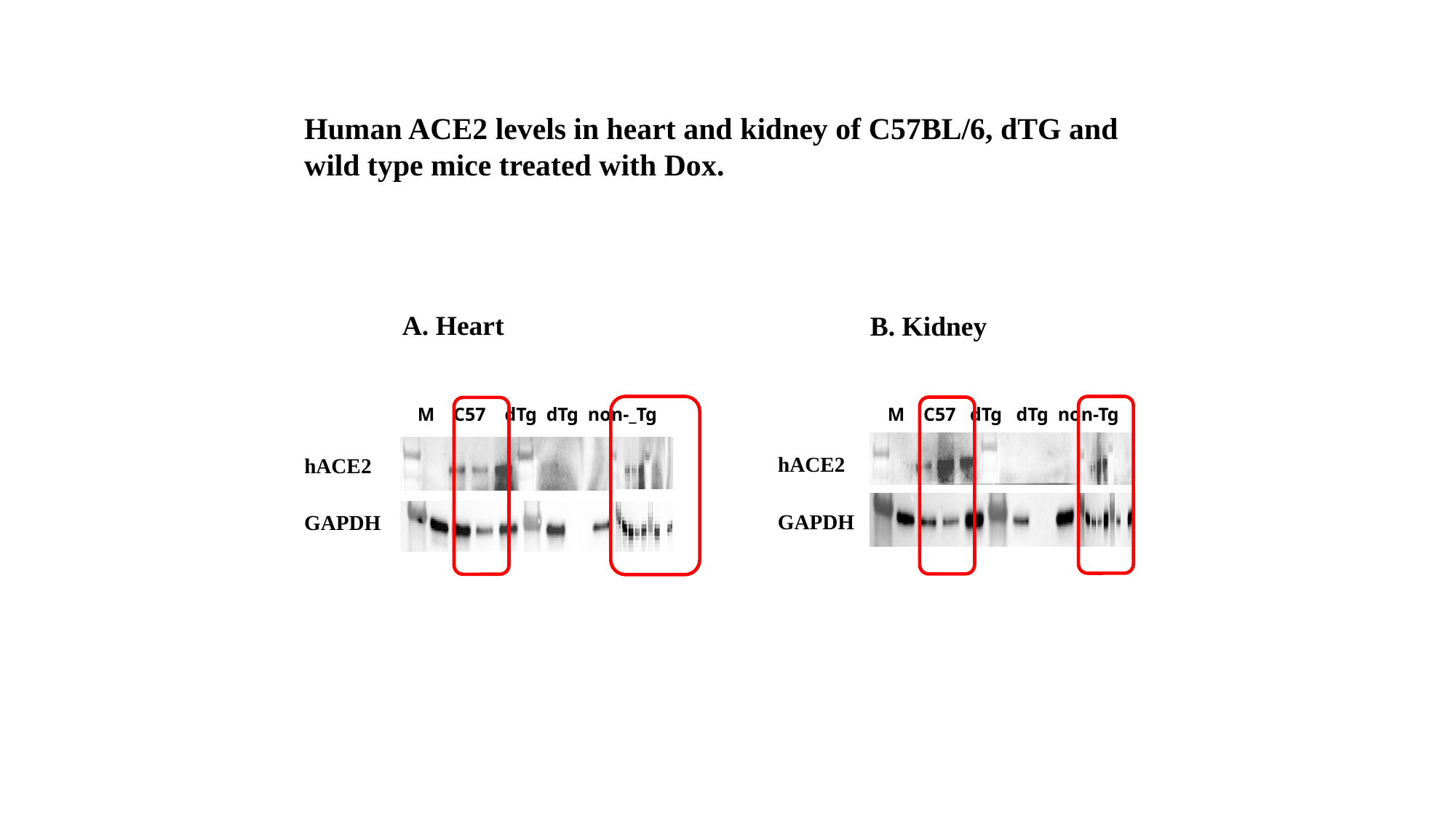

Human ACE2 levels in heart and kidney of C57BL/6, dTG and wild type mice treated with Dox.
A. Heart
M C57 dTg dTg non-_Tg
B. Kidney
M C57 dTg dTg non-Tg
hACE2
hACE2
GAPDH
GAPDH
