# Supplementary material for: Characterization of changes in global gene expression in the hearts and kidneys of transgenic mice overexpressing human angiotensin-converting enzyme 2
Source: Lab Anim Res. 2020 Jul 29;36:23. doi: 10.1186/s42826-020-00056-y (PMC7387885; doi:10.1186/s42826-020-00056-y)
Supplement: Supplementary file 3 — Additional file 3. [file 42826_2020_56_MOESM3_ESM.pptx]

## Slide 1
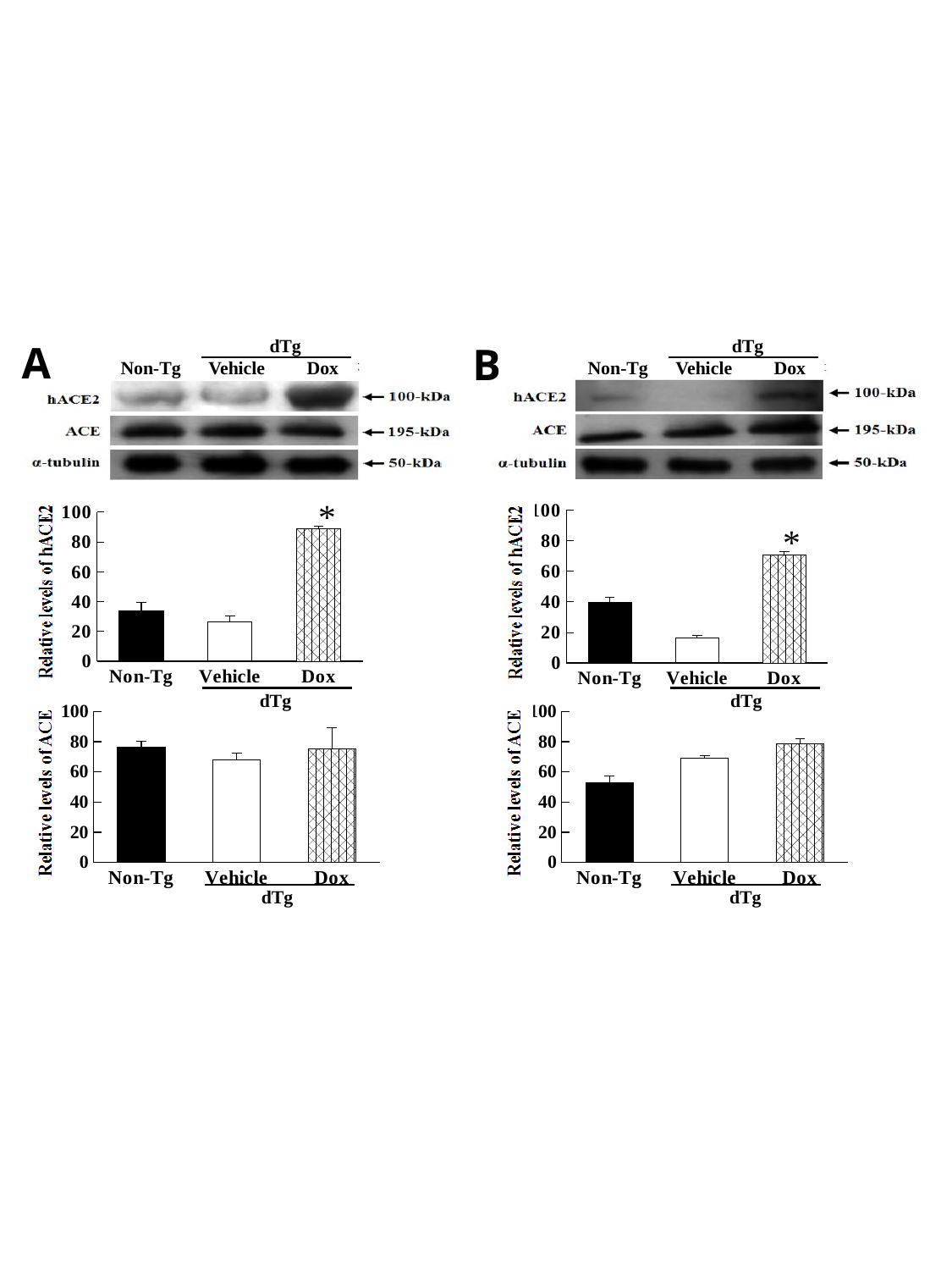

dTg
Non-Tg Vehicle Dox
A
*
### Chart
| Category | |
|---|---|
| Non-Tg | 33.71 |
| Vehicle | 26.3 |
| Dox | 89.05 |dTg
### Chart
| Category | |
|---|---|
| Non-Tg | 76.19 |
| Vehicle | 67.83 |
| Dox | 75.12 |dTg
 dTg
Non-Tg Vehicle Dox
B
### Chart
| Category | |
|---|---|
| Non-Tg | 39.6 |
| Vehicle | 16.24 |
| Dox | 70.65 |dTg
*
### Chart
| Category | |
|---|---|
| Non-Tg | 52.72 |
| Vehicle | 69.31 |
| Dox | 78.66 |dTg
